# Disinfection methods for preventing COVID-19 infections in healthcare settings: A rapid review

**DOI:** 10.4102/jphia.v16i2.588

**Published:** 2025-02-25

**Authors:** Joseph Okebe, Atana Ewa, Ememobong Aquaisua, Obasesam A. Ikpi, Ella Olughu, Ebere C. Chukwuemelie, Chukwudi Oringanje, Tochi Okwor, Martin Meremikwu

**Affiliations:** 1 Department of International Public Health, Liverpool School of Tropical Medicine, Liverpool, United Kingdom; 2 Institute of Tropical Diseases Research and Prevention, University of Calabar Teaching Hospital, Calabar, Nigeria; 3 Department of Pediatrics, University of Calabar Teaching Hospital, Calabar, Nigeria; 4 Department of Health and Demographic Surveillance System, University of Calabar, Calabar, Nigeria; 5 Nigeria Centre for Disease Control and Prevention, Abuja, Nigeria

**Keywords:** prevention, disinfection, SARS-CoV-2, healthcare, COVID-19

## Abstract

**Background:**

Disinfectant sprays and wipes reduce the risk of infection from contaminated surfaces and materials in healthcare facilities. To support guideline updates, evidence on surface disinfection against the severe acute respiratory syndrome coronavirus 2 (SARS-CoV-2) infection are needed.

**Aim:**

This study aims to compare the effect of disinfection by spraying or wiping on the risk of human infections in healthcare facilities providing coronavirus disease 2019 (COVID-19) services.

**Setting:**

Healthcare settings providing care for patients with COVID-19 or where exposure risk to COVID-19 is high.

**Method:**

We searched the Central Register of Controlled Trials (CENTRAL) and Cochrane Database of systematic review; PubMed, EMBASE and EPOC databases from 01 January 2020 to 31 August 2022. Results were screened for eligibility, the risk of bias in included studies assessed, and the certainty of evidence defined using GRADE^®^.

**Results:**

Three observational studies were included. Two studies reporting proportion of surfaces with residual contamination, showed contrasting results with spraying more effective (0%, [*n* = 0/39] vs. 25.6% [*n* = 23/90]) in one study but less effective (25.0% [*n* = 12/48] vs. 48.2% [*n* = 13/27]) in the other. The third study reported higher reductions from wiping (88.0%) compared to spraying (15.1%). The risk of bias ranged from moderate to serious and the certainty of the evidence was very low. No study reported a direct effect on the risk of infection in humans.

**Conclusion:**

Both spraying and wiping methods may protect against SARS-CoV-2 infections indirectly by reducing residual surface contamination.

**Contribution:**

The use of both methods of disinfection in cleaning protocols indirectly reduces residual surface contamination.

## Introduction

COVID-19 remains an important public health challenge globally with over 650 million cases and about 6.5 million deaths reported globally as on October 2022.^1^ Transmission is mainly airborne through droplets and aerosolised particles containing the severe acute respiratory syndrome coronavirus 2 (SARS-CoV-2).^2^ The risk of transmission is associated with proximity to an infectious source hence may be higher in poorly ventilated, indoor spaces and with prolonged exposure.^3,4^ Although transmission is possible from contact with contaminated surfaces and materials, this has relatively lower risk but could be an important source in high-risk settings such as health facilities, schools and transport hubs.^3,4,5^ Reports of SARS-CoV-2 outbreaks in healthcare facilities suggest the potential for fomite transmission in healthcare settings.^5,6,7^

In healthcare settings there is a substantial risk of indirect infection from contaminated surfaces, equipment and shared spaces.^6,8,9,10^ Infection prevention and control (IPC) guidelines address this risk through guidance on measures such as social distancing, disinfection, use of face covering and other personal protective equipment.^11^

Disinfection of surfaces, equipment and materials is an integral part of IPC protocols in healthcare settings.^12,13^ These protocols contain information on best practice on how to reduce the risk of infection to both staff and patients. They provide details on cleaning procedures, the type of disinfectant and methods for application on specific surfaces. Generally, disinfectants are applied either as direct spraying on surfaces or incorporated into wiping or scrubbing materials used in manual cleaning of surfaces and equipment. The goal is to optimise the microbicidal action of the agents by taking into account the type of surface, contact time, quantity of disinfectant applied and type of applicator used.^14^

Disinfectant products are labelled for use based on their treatment time (time needed to achieve a threshold microbicidal action) with regulators recommending a contact time of ≥ 1 min at the proper use dilution for disinfecting noncritical medical equipment and surfaces.^15,16,17^ The disinfectant action of sprays is directly related to its treatment time while the effect of wiping and brushing methods of applying disinfectants involves the additional effect from the process of manual removal action.

Many public health authorities have issued updated guidance on cleaning and disinfection protocols in the wake of the COVID-19 pandemic.^18,19^ With the increased usage of disinfectants, there have been questions about the relative importance of spraying and wiping methods in healthcare settings.^20,21^ This review assesses the available evidence on the effectiveness of spraying and wiping methods for disinfecting surfaces in healthcare settings providing care to patients with COVID-19.

## Methods

### Criteria for considering studies for this review

We included comparative studies conducted in healthcare facilities involving use of disinfectants by sprays or wiping action. Because of the rapidly evolving nature of the evidence on the pandemic, we prioritised studies for inclusion based on the study design and methodological rigour. In the first instance, we considered individual, or cluster randomised controlled trials and where these were not available, we included other types of studies provided they included at least one comparative arm involving the spraying method for disinfection. We searched the Central Register of Controlled Trials (CENTRAL), Cochrane Database of Systematic Review; PubMed, EMBASE and EPOC (The Effective Practice and Organisation of Care) for the period 01 January 2020 – 31 August 2022. We restricted the search to studies conducted in healthcare settings or where samples were drawn from healthcare settings as this would provide direct evidence to the review question. We excluded simulation studies conducted in the research laboratories as well as studies on bacterial decontamination. We also checked the reference lists of retrieved studies for additional reports of relevant studies. No language restrictions were applied (Table 1).

**TABLE 1:** Search strategy and output.

Search number	Query description
1.	exp Health facilities/ or exp hospitals/ or exp Nursing Homes/ or Homes for the Aged/ or assisted living facilities/
2.	exp Health Personnel/ or exp Health Occupations/
3.	((medical or health or healthcare) adj3 (facility or facilities or centre* or center* or office* or setting*)).ti,ab,kf.
4.	((medical or health care or healthcare) adj (worker* or staff or employee* or personnel or occupation*)).ti,ab,kf.
5.	(hospital* or ward* or physician* or doctor* or surgeon* or surgical or surger* or dentist* or dental or nurs* or infirmar* or hospice* or Clinic or Clinics or ‘Old Age Homes’ or ‘Old Age Home’).ti,ab,kf.
6.	((longterm care or long term care) adj3 (home* or facility or facilities or residence* or housing* or setting*)).ti,ab,kf.
7.	Pharmacies/ not Health facilities/
8.	(Caregivers/ or Pharmacists/) not (Health Personnel/ or Health Occupations/)
9.	or/1–6
10.	or/7–8
11.	9 not 10
12.	Disinfection/ or exp Disinfectants/ or exp Detergents/ or Fumigation/ or Decontamination/
13.	exp chlorine compounds/ or hypochlorous acid/ or sodium hypochlorite/
14.	Quaternary Ammonium Compounds/ or Bleaching Agents/ or Hydrogen Peroxide/
15.	exp ethanol/ or exp ethanolamines/ or 2-Propanol/
16.	methylene blue/
17.	((sanitation or sanitary or housekeep* or janitor*) adj3 (worker* or staff or employee* or personnel or occupation* or team* or department*)).ti,ab,kf.
18.	(brush or brushing or clean* or disinfect* or decontaminat* or fogging or fog or fogger* or fumigat* or mist or misting or sanitis* or sanitiz* or scrub or scrubbing or spray* or wipe or wiping).ti,ab,kf.
19.	(detergent* or soap* or methylene blue or ethanol or bleach* or chlorin* or hypochlorit* or hydrogen peroxide or glutaraldehyde or formaldehyde or aldehyde or dihydrogen dioxide or hydrogen dioxide or hydroperoxide or quaternary ammonium or quaternary bisammonium or quaternized amine or hypochlorous acid).ti,ab,kf.
20.	(antiseptic* or iodine or ethanolamin* or isopropanol or isopropyl or propanol or 2propanol).ti,ab,kf.
21.	or/12–20
22.	(cisplatin/ or exp Hydrochloric acid/) not (chlorine compounds/ or hypochlorous acid/ or sodium hypochlorite/)
23.	21 not 22
24.	(hand wash* or hand hygiene or hand sanitisation or hand sanitisation or hand disinfection or hand decontamination or skin disinfection or skin decontamination or skin wash* or skin hygiene or skin sanitisation or skin sanitisation or hands or eczema* or mouth or oral* or nose or handwash* or skinwash* or mouthwash* or nosewash* or nasal* or topical* or ultraviolet or ultra violet or UV or UVC or UVGI or actinic).ti.
25.	((PPE or ‘personal protective equipment’ or mask* or facemask* or respirator or respirators or N95 or N99 or KN95 or FFP2 or FFP3 or ‘Filtering face piece’ or ‘Filtering face pieces’ or ‘Filtering facepiece’ or ‘Filtering facepieces’ or ‘respiratory protective device’ or ‘respiratory protective devices’) adj2 (disinfect* or decontaminat* or sanitiz* or sanitis* or clean* or wash*)).ti.
26.	((PPE or ‘personal protective equipment’ or mask* or facemask* or respirator or respirators or N95 or N99 or KN95 or FFP2 or FFP3 or ‘Filtering face piece’ or ‘Filtering face pieces’ or ‘Filtering facepiece’ or ‘Filtering facepieces’ or ‘respiratory protective device’ or ‘respiratory protective devices’) not (surface* or touch* or high touch or high traffic or environment* or material* or object or objects or building* or floor* or counter* or bed or beds or equipment* or room or rooms or space* or door* or chair* or wheelchair* or washroom* or bathroom* or elevator* or toilet* or contact time* or wood* or plastic* or metal* or fog* or fumigat* or mist or misting or spray*)).ti.
27.	24 or 25 or 26
28.	23 not 27
29.	limit 28 to covid-19
30.	11 and 29
31.	limit 30 to (meta analysis or ‘systematic review’)
32.	randomised controlled trial.pt.
33.	controlled clinical trial.pt.
34.	randomised.ab.
35.	placebo.ab.
36.	randomly.ab.
37.	trial.ab.
38.	groups.ab.
39.	or/32–38
40.	exp animals/ not humans.sh.
41.	39 not 40 [*adapted from the Cochrane HSSS RCT filter*]
42.	30 and 41
43.	exp cohort studies/ or exp epidemiologic studies/ or exp clinical trial/ or exp evaluation studies as topic/ or exp statistics as topic/
44.	((time and factors) or program or survey* or ci or cohort or comparative stud* or evaluation studies or follow-up*).mp.
45.	(control and (group* or study)).mp.
46.	or/43–45
47.	(animals/ not humans/) or comment/ or editorial/ or exp review/ or meta analysis/ or consensus/ or exp guideline/
48.	hi.fs. or case report.mp.
49.	or/47–48
50.	46 not 49 [*NRS filter by Waffenschmidt et al. 2020*]
51.	30 and 50
52.	31 or 42 or 51
53.	exp Animals/not Humans/
54.	52 not 53
55.	(comment or editorial or newspaper article).pt.
56.	54 not 55
57.	((sanitation or sanitary or housekeep* or janitor*) adj (worker* or staff or employee* or personnel or occupation* or team* or department*)).ti,ab,kw.
58.	(clean* or disinfect* or decontaminat* or sanitis* or sanitiz*).ti,ab,kw.
59.	(mist or misting or scrub or scrubbing or spray* or wipe or wiping).ti,ab. not scrub typhus.mp.
60.	(brush or brushing or fogging or fogger* or fumigat*).ti,ab.
61.	(detergent* or soap* or methylene blue or ethanol or bleach* or chlorin* or hypochlorit* or hydrogen peroxide or glutaraldehyde or formaldehyde or aldehyde or dihydrogen dioxide or hydrogen dioxide or hydroperoxide or quaternary ammonium or quaternary bisammonium or quaternized amine or hypochlorous acid).ti,ab,kw.
62.	(antiseptic* or iodine or ethanolamin* or isopropanol or isopropyl or propanol or 2propanol).ti,ab,kw.
63.	(hand* or skin* or eczema* or mouth* or oral* or nose* or nasal* or intranasal* or inhal* or nebuli* or throat* or gargl* or topical* or ultraviolet or ultra violet or UV or UVC or UVGI or actinic).ti,kw.
64.	(PPE or ‘personal protective equipment’ or mask* or facemask* or respirator or respirators or N95 or N99 or KN95 or FFP2 or FFP3 or ‘Filtering face piece’ or ‘Filtering face pieces’ or ‘Filtering facepiece’ or ‘Filtering facepieces’ or ‘respiratory protective device’ or ‘respiratory protective devices’).ti,kw. or exp mask/ or exp respiratory protection/ or protective equipment/
65.	drinking/ or exp alcoholism/ or exp drug dependence/ or exp addiction/
66.	63 or 64 or 65
67.	disinfection/
68.	decontamination/
69.	fumigation/
70.	exp disinfectant agent/ or 2 propanol/ or alcohol/ or formaldehyde/ or glutaraldehyde/ or hypochlorite sodium/ or iodine/
71.	detergent/
72.	chlorine/ or chlorine derivative/
73.	hypochlorous acid/
74.	quaternary ammonium derivative/ or benzalkonium/ or benzalkonium chloride/ or tetrylammonium/
75.	bleaching agent/ or hydrogen peroxide/
76.	ethanolamine derivative/ or ethanolamine/
77.	methylene blue/
78.	or/57–62,67–77
79.	78 not 66
80.	limit 79 to covid-19
81.	exp health care facility/ or assisted living facility/ or exp hospital/ or nursing home/
82.	home for the aged/
83.	exp health care personnel/
84.	medical profession/
85.	((medical or health or healthcare) adj3 (facility or facilities or centre* or center* or office* or setting*)).ti,ab,kw.
86.	((medical or health care or healthcare) adj (worker* or staff or employee* or personnel or occupation*)).ti,ab,kw.
87.	(hospital* or physician* or doctor* or surgeon* or surgical or surger* or dentist* or dental or nurs* or infirmar* or hospice* or Clinic or Clinics or ‘Old Age Homes’ or ‘Old Age Home’).ti,ab,kw.
88.	((longterm care or long term care) adj3 (home* or facility or facilities or residence* or housing* or setting*)).ti,ab,kw.
89.	exp ‘pharmacy (shop)’/ not health care facility/
90.	(exp pharmacist/ or exp health educator/) not (paramedical personnel/ or health care personnel/ or health practitioner/ or paramedical profession/ or medical profession/)
91.	or/81–88
92.	89 or 90
93.	91 not 92
94.	80 and 93
95.	limit 94 to ‘systematic review’
96.	randomised controlled trial/ or controlled clinical trial/ or randomisation/ or exp intermethod comparison/ or double-blind procedure/
97.	human experiment/
98.	(random$ or crossover or cross over or volunteer or volunteers or placebo or allocated or assigned or (open adj label)).ti,ab.
99.	(compare or compared or comparison).ti.
100.	((evaluated or evaluate or evaluating or assessed or assess) and (compare or compared or comparing or comparison)).ab.
101.	((double or single or doubly or singly) adj (blind or blinded or blindly)).ti,ab.
102.	parallel group$1.ti,ab.
103.	((assign$ or match or matched or allocation) adj5 (alternate or group$1 or intervention$1 or patient$1 or subject$1 or participant$1)).ti,ab.
104.	(controlled adj7 (study or design or trial)).ti,ab.
105.	trial.ti.
106.	or/96–105
107.	(Systematic review not (trial or study)).ti.
108.	(random cluster adj3 sampl$).ti,ab.
109.	(review.ab. and review.pt.) not trial.ti.
110.	(databases adj4 searched).ab.
111.	‘we searched’.ab. and (review.ti. or review.pt.)
112.	‘update review’.ab.
113.	Random field$.ti,ab.
114.	(nonrandom$ not random$).ti,ab.
115.	(random$ adj sampl$ adj7 (cross section$ or questionnaire$1 or survey$ or database$1)).ti,ab. not (comparative study/ or controlled study/ or randomised controlled.ti,ab. or randomly assigned.ti,ab.)
116.	Cross-sectional study/ not (randomised controlled trial/ or controlled clinical trial/ or controlled study/ or randomi?ed controlled.ti,ab. or control group$1.ti,ab.)
117.	(((case adj control$) and random$) not randomi?ed controlled).ti,ab.
118.	(rat or rats or mouse or mice or swine or porcine or murine or sheep or lambs or pigs or piglets or rabbit or rabbits or cat or cats or dog or dogs or cattle or bovine or monkey or monkeys or trout or marmoset$1).ti. and animal experiment/
119.	Animal experiment/ not (human experiment/ or human/)
120.	(‘cochrane database of systematic reviews’ or ‘cochrane database of systematic reviews online’).jn.
121.	or/107–120
122.	106 not 121 [*Cochrane Embase RCT filter 2022 revision (Glanville et al. 2019)*]
123.	94 and 122
124.	Clinical article/ or controlled study/ or major clinical study/ or prospective study/ or cohort.mp. or compared.mp. or groups.mp. or multivariate.mp. [*NRS filter by Furlan et al. 2006 for Embase*]
125.	94 and 124
126.	95 or 123 or 125
127.	exp animal/ not exp human/
128.	126 not 127
129.	editorial.pt.
130.	128 not 129
131.	1 or 2 or 85 or 86 or 87 or 88
132.	131 not 10
133.	12 or 13 or 14 or 15 or 16 or 57 or 58 or 59 or 60 or 61 or 62
134.	133 not 27
135.	(nCoV* or 2019nCoV or 19nCoV or COVID19* or COVID or SARS-COV-2 or SARSCOV-2 or SARS-COV2 or SARSCOV2 or coronavirus* or corona virus* or betacoronavirus* or CoV or HCoV).mp.
136.	limit 135 to yr = ‘2019 –Current’ [*COVID-19 filter adapted from CADTH Search Strings*]
137.	132 and 134 and 136
138.	56 use medal
139.	130 use emczd
140.	137 use cctr
141.	137 use coch
142.	138 or 139 or 140 or 141
143.	remove duplicates from 142

Note: Embase Classic + Embase 1947 to 02 September 2022, Ovid MEDLINE(R) ALL 1946 to 02 September 2022, EBM Reviews – Cochrane Central Register of Controlled Trials July 2022, EBM Reviews – Cochrane Database of Systematic Reviews 2005 to 31 August 2022.

### Participants, interventions and outcomes

The primary outcome in the review was the risk of SARS-CoV-2 infection in humans (all definitions for infection in humans described by the study authors). Other outcomes were laboratory-confirmed SARS-CoV-2 on surfaces and materials, residual surface contamination following disinfection and reported adverse effects from the decontamination method. We included studies that compared spraying with wiping methods for disinfection of surfaces, materials and equipment. Wiping methods included brushing, scrubbing tools, disinfectant-embedded materials such as wipes or towels on surfaces and materials. We excluded descriptive studies on surface contamination that did not involve an assessment of a cleaning intervention that included spraying and wiping.

### Screening and data extraction

**FIGURE 1:**
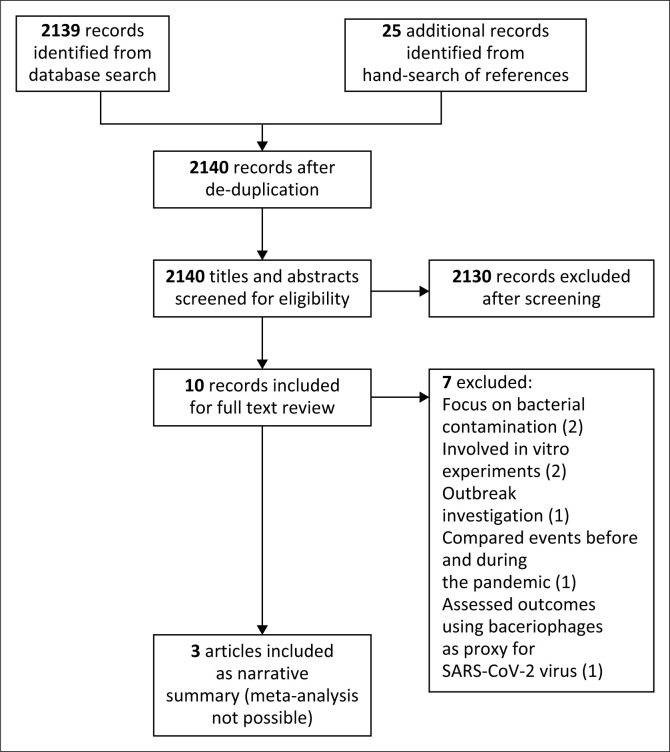
Preferred Reporting Items for Systematic Reviews and Meta-Analyses (PRISMA) flowchart showing article screening and inclusion in the review.

Two review authors independently applied our eligibility criteria to screen the titles and abstracts from the retrieved search output after de-duplication. Where multiple articles based on the same study were seen, they were distinguished by adding a suffix to the publication year. The full text of studies that met the initial criteria were retrieved for a more detailed eligibility screen by two independent reviewers. Any discrepancies in selection of studies were resolved by discussion among the review team. No changes were made to the protocol in terms of eligibility and selection criteria. The result of the screening process is presented in a Preferred Reporting Items for Systematic reviews and Meta-Analyses (PRISMA) flowchart (Figure 1).

For each included study, we extracted background information on the location and context of the study and any demographic information if available (e.g., type of health facility, availability of cleaning protocol). We recorded information on the number of participants and surfaces included and analysed in each arm or group. We extracted data on the review outcomes and documented other outcomes reported by study authors but not related to the review. Dichotomous outcomes are reported as proportions and continuous outcomes reported as means or medians in each arm or group.

### Risk of bias assessment in included studies

We assessed the risk of bias in the included studies using the risk of bias in non-randomised studies of interventions (ROBINS-I) tool. The tool assesses potential bias for key review outcomes across risk domains: confounding, selection of participants, classification of the intervention, deviations from intended intervention, missing data, measurement outcomes and selective reporting. Each domain is assigned a score with an overall risk score assigned for each study.^22^ The results are presented in a ‘Risk of bias’ assessment table.

### Data analysis and assessment of the certainty of the evidence

Data were extracted onto piloted forms designed using Microsoft Excel. Two authors independently extracted data with consistency checks done by a third author. Outcomes were pooled together where feasible. Because of substantial clinical and methodological heterogeneity across the studies, a meta-analysis was not possible. Therefore results are presented as a narrative summary. The certainty of evidence was assessed following the protocol for grading of recommendations, assessment, development and evaluations (GRADE^®^).^23^ This is a transparent framework for developing and presenting summaries of evidence and provides a systematic approach for supporting clinical practice recommendations.

### Ethical considerations

This article followed all ethical standards for research without direct contact with human or animal subjects.

## Results

### Results of the search

The search returned 2164 articles: 2139 from the database search and 25 records of studies from hand search of references from a review.^24^ After removing duplicate publications, we screened the titles and abstracts of 2140 records from which 10 articles were selected for full-text assessment. Of these 10, three studies met the eligibility criteria and were included in the review. A full description of the studies is presented in tabular form (Table 2). The process of screening and selection is presented in a PRISMA flow diagram (Figure 1). The excluded studies and reasons for exclusion are listed in Table 3.

**TABLE 2:** Characteristics of included studies.

Study ID	Methods	Surfaces	Interventions	Outcomes	Notes
Kim et al. 2020^25^	Investigated air and environmental contamination in rooms used to manage COVID-19 patients in four hospitals. Cross-sectional sampling from surfaces in rooms – 1 day, 3 days, 5 days and 7 days after admission.	Different room types in four hospitals. Background on some patients admitted in the rooms provided; eight participants in total.	Disinfection before admission and after discharge; some rooms had protocols that included daily wiping verses twice daily spray with disinfectant	Air and environmental (surface) contamination by detectable viral RNA detected by RT-PCR	Room conditions differed by hospital. Hospitals A and B had seven and five designated airborne infection isolation rooms (AIIRs), respectively, with a minimum of 15 air changes per hour. Hospital C is a designated COVID-19 hospital with isolation rooms without negative air pressure. In Hospital D, patients are grouped in common rooms without negative air pressure.
Lesho et al. 2022^26^	Evaluated the effectiveness of daily, enhanced terminal and contingency-based cleaning strategies in an acute care facility in hospitals (ACH) and in long-term care wards in a health facility (LTCF), using SARS-CoV-2 RT-PCR and adenosine triphosphate (ATP) assays.	Eight stationary near-patient, high touch surfaces.	Terminal cleaning (wiping) followed by ultraviolet light or electrostatic spray, a combination of both or room fogging	Environmental contamination with SAR-CoV-2 RNA	Two sampling schemes were applied: random and controlled. In the random subgroup, stationary surface types sampled at random timepoints in-between cleaning rounds while in the controlled group, the same eight stationary surface types sampled in the random assessment were sampled immediately before and after terminal cleaning that involved surface wiping. The controlled group also involved additional cleaning intervention applied as enhancements over surface wiping. The review focused on data from the controlled subgroups from both the ACH and LTCF. The data from these subgroups are analysed differently, hence presented separately.
Lugo et al. 2021^27^	Aim was to determine the efficacy of four disinfectants in the hospital environment when applied with local cleaning and disinfection standards. Observational study.	Surfaces and materials in rooms/sections in two hospitals.	Four different disinfection methods: disinfection by wiping, spraying, electromagnetic methods, UV light treatment	Change in proportion and volume of contamination/contaminants	(1) Electrolysed super-oxidation solution with neutral pH at 0.004% active Cl, (2) solution formulated at neutral pH (6.2–7.3) with active substances containing activated high-power chlorine oxidants, such as hypochlorous acid 75%, hypochlorous ion 15%, chlorine dioxide 8% and ozone 2% (3) UV light rays (240 nm), (4) wet wipes with 0.5% w/w hydrogen peroxide-based disinfectant.

Note: Please see the full reference list of the article Okebe J, Ewa A, Aquaisua E, et al. Disinfection methods for preventing COVID-19 infections in healthcare settings: A rapid review. J Public Health Africa. 2025;16(2), a588. https://doi.org/10.4102/jphia.v16i2.588, for more information.

COVID-19, coronavirus disease 2019; SARS-CoV-2, severe acute respiratory syndrome coronavirus 2; RNA, ribonucleic acid; RT-PCR, reverse transcription polymerase chain reaction; UV, ultraviolet; w/w, weight per weight; pH, potential of hydrogen (acidity or basicity); CI, chloride; ID, identification.

**TABLE 3:** Summary characteristics of excluded studies.

Study ID	Reason for exclusion
Bailey et al. 2021^29^	Four essential oil used as spray disinfectant, inactivation of bacteria and viruses. Lab-based study using a SARS-CoV-2 virus surrogate.
Balter et al. 2021^30^	Focus on bacterial contamination.
Bigham et al. 2022^31^	Focus on bacterial contamination.
Campos et al. 2021^32^	In vitro plaque assay using assay plates and synthetic pig skin.
Chen et al. 2021^33^	This is a comparative study between pre-COVID-19 and post-COVID-19 era.
Cheng et al. 2021^34^	Outbreak investigation report.
Viana et al. 2022^35^	The study examined the effectiveness of various disinfectants using relative light units with a special swab containing the enzyme luciferin sulfotransferase that is activated by contact with organic matter as the measure of effectiveness.

Note: Please see the full reference list of the article Okebe J, Ewa A, Aquaisua E, et al. Disinfection methods for preventing COVID-19 infections in healthcare settings: A rapid review. J Public Health Africa. 2025;16(2), a588. https://doi.org/10.4102/jphia.v16i2.588, for more information.

Lab, laboratory; ID, identification; SARS-CoV-2, severe acute respiratory syndrome coronavirus 2; COVID-19, coronavirus disease 2019.

### Description of studies: Design, population, interventions and outcomes

The three included studies were conducted in hospitals in South Korea,^25^ United States (US)^26^ and Mexico.^27^ The study setting ranged from specific surfaces in single to multi-occupancy rooms to materials and equipment in treatment and examination suites. The design and setting were very different between the studies. In the US study, all surfaces were disinfected by wiping and then compared for the relative effect of additional methods that included spraying.^26^ In the hospital in South Korea, the study was conducted across four hospitals using a range of intervention approaches; however, only two of the four hospitals implemented spraying and wiping and these groups were included in the review.^25^ In the hospital in US, samples were from multiple surfaces in the same location^26^ while in the other two studies, sampling was collected from multiple surfaces and locations in the same health facility. All studies report adherence to cleaning protocols but did not provide details of the protocols. Only the study in South Korea described the patients who occupied the rooms where the study was conducted. However, there was no information on the risk of SARS-CoV-2 infection in patients or healthcare workers.

The effectiveness of the disinfection methods was reported as a change in viral ribonucleic acid (RNA) concentration measured in relative light units in one study^27^ and as the proportion of sampled surfaces with detectible RNA using a threshold Ct value of 35 or less after cleaning in the other two studies.^25,26^ Data were presented for the number of samples taken across the surfaces. The studies present crude results without any adjustments in effect measures and no information was provided to allow for additional analysis in the review. Therefore a meta-analysis was not feasible and results are presented as a narrative summary.

### Risk of bias assessment

The overall risk of bias was rated as moderate in two studies^25,26^ and serious in one study^27^ although risks varied between the studies across specific domains. A summary of the assessment is presented in Table 4. There were differences in the location and type of surfaces where samples were collected. All studies applied a cleaning protocol; however, the details of these protocols were not provided and hence alignment and consistency with protocols could not be assessed. Two studies reported using an additional cleaning protocol over what was routinely provided.^26,27^ It is probable that investigators who analysed the samples were aware of the source of the samples; however, no information was provided to determine if they were blinded to source of the samples. No statistical assessment for heterogeneity was possible in the review.

**TABLE 4:** Risk of bias in included studies assessed using the risk of bias in a non-randomised studies of interventions tool.

Signalling questions	Kim et al. 2020^25^	Lesho et al. 2022^26^	Lugo et al. 2021^27^
**Bias because of confounding**			
1.1. Is there potential for confounding of the effect of intervention in this study?	Yes	No	Yes
**If N/PN to 1.1:** The study can be considered to be at low risk of bias because of confounding and no further signalling questions need be considered	-	-	-
**If Y/PY to 1.1:** Determine whether there is a need to assess time-varying confounding:	-	-	-
1.2. Was the analysis based on splitting participants’ follow-up time according to intervention received?	No	-	No
**If N/PN,** answer questions relating to baseline confounding (1.4–1.6)	-	-	-
**If Y/PY,** go to question 1.3.	-	-	-
1.3. Were intervention discontinuations or switches likely to be related to factors that are prognostic for the outcome?	-	-	-
**If N/PN,** answer questions relating to baseline confounding (1.4–1.6)	-	-	-
**If Y/PY,** answer questions relating to both baseline and time-varying confounding (1.7 and 1.8)	-	-	-
**Questions relating to baseline confounding only**	-	-	-
1.4. Did the authors use an appropriate analysis method that controlled for all the important confounding domains?	No information	-	No
1.5. **If Y/PY to 1.4:** Were confounding domains that were controlled for measured validly and reliably by the variables available in this study?	-	-	-
1.6. Did the authors control for any post-intervention variables that could have been affected by the intervention?	No information	-	No information
**Questions relating to baseline and time-varying confounding**	-	-	-
1.7. Did the authors use an appropriate analysis method that controlled for all the important confounding domains and for time-varying confounding?	No	-	No information
1.8. **If Y/PY to 1.7:** Were confounding domains that were controlled for measured validly and reliably by the variables available in this study?	-	-	-
**Risk of bias judgement**	Serious	Low	Serious
**Bias in selection of participants into the study**			
2.1. Was selection of participants into the study (or into the analysis) based on participant characteristics observed after the start of intervention?	No	Probably no	No
**If N/PN to 2.1:** Go to 2.4	-	-	-
2.2. **If Y/PY to 2.1:** Were the post-intervention variables that influenced selection likely to be associated with intervention?	-	-	-
2.3. **If Y/PY to 2.2:** Were the post-intervention variables that influenced selection likely to be influenced by the outcome or a cause of the outcome?	-	-	-
2.4. Do start of follow-up and start of intervention coincide for most participants?	Yes	Yes	Probably yes
2.5. **If Y/PY to 2.2 and 2.3, or N/PN to 2.4:** Were adjustment techniques used that are likely to correct for the presence of selection biases?	-	-	-
**Risk of bias judgement**	Moderate	Low	Low
**Bias in classification of interventions**			
3.1. Were intervention groups clearly defined?	Yes	Yes	No
3.2. Was the information used to define intervention groups recorded at the start of the intervention?	No information	No information	Probably no
3.3. Could classification of intervention status have been affected by knowledge of the outcome or risk of the outcome?	No	Yes	Probably yes
**Risk of bias judgement**	Low	Moderate	Serious
Optional: What is the predicted direction of bias because of classification of interventions?	-	-	-
**Bias because of deviations from intended interventions**			
**If your aim for this study is to assess the effect of assignment to intervention, answer questions 4.1 and 4.2**	-	-	-
4.1. Were there deviations from the intended intervention beyond what would be expected in usual practice?	No	-	Probably yes
4.2. **If Y/PY to 4.1:** Were these deviations from intended intervention unbalanced between groups *and* likely to have affected the outcome?	Not applicable	-	Yes
**If your aim for this study is to assess the effect of starting and adhering to intervention, answer questions 4.3 to 4.6**	-	-	-
4.3. Were important co-interventions balanced across intervention groups?	-	Yes	-
4.4. Was the intervention implemented successfully for most participants?	-	No information	-
4.5. Did study participants adhere to the assigned intervention regimen?	-	No information	-
4.6. **If N/PN to 4.3, 4.4 or 4.5:** Was an appropriate analysis used to estimate the effect of starting and adhering to the intervention?	-	No information	-
**Risk of bias judgement**	Low	Serious	Serious
**Bias because of missing data**			
5.1. Were outcome data available for all, or nearly all, participants?	Yes	Yes	Probably yes
5.2. Were participants excluded because of missing data on intervention status?	Probably no	No	No information
5.3. Were participants excluded because of missing data on other variables needed for the analysis?	No	No information	No information
5.4. **If PN/N to 5.1, or Y/PY to 5.2 or 5.3:** Are the proportion of participants and reasons for missing data similar across interventions?	-	-	-
5.5. **If PN/N to 5.1, or Y/PY to 5.2 or 5.3:** Is there evidence that results were robust to the presence of missing data?	-	-	-
**Risk of bias judgement**	Low	Moderate	Moderate
**Bias in measurement of outcomes**			
6.1. Could the outcome measure have been influenced by knowledge of the intervention received?	Probably yes	Probably yes	Yes
6.2. Were outcome assessors aware of the intervention received by study participants?	No information	No information	Probably yes
6.3. Were the methods of outcome assessment comparable across intervention groups?	Yes	Yes	Yes
6.4. Were any systematic errors in measurement of the outcome related to intervention received?	No	-	No
**Risk of bias judgement**	Moderate	Moderate	Serious
Optional: What is the predicted direction of bias because of measurement of outcomes?	-	-	-
**Bias in selection of the reported result**			
Is the reported effect estimate likely to be selected, on the basis of the results, from …	-	-	-
7.1. … multiple outcome *measurements* within the outcome domain?	No	No	Yes
7.2. … multiple *analyses* of the intervention-outcome relationship?	No	No	No
7.3. … different *subgroups*?	No	Probably yes	Yes
**Risk of bias judgement**	Low	Moderate	Serious

**Overall risk of bias judgement**	**Moderate**	**Moderate**	**Serious**

Note: Please see the full reference list of the article Okebe J, Ewa A, Aquaisua E, et al. Disinfection methods for preventing COVID-19 infections in healthcare settings: A rapid review. J Public Health Africa. 2025;16(2), a588. https://doi.org/10.4102/jphia.v16i2.588, for more information.

N, no; Y, yes; PN, probably no; PY, probably yes.

### Effectiveness of surface decontamination

**FIGURE 2:**
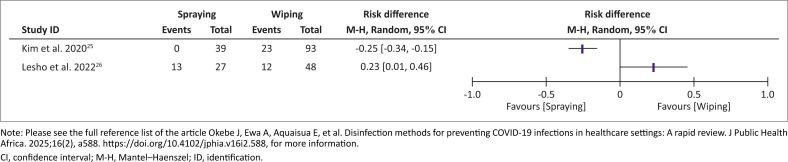
Summary of effect of spraying versus wiping method on residual surface contamination.

Studies reported on the effectiveness of surface decontamination, measured by residual contamination after cleaning, showed divergent results. In one study, spraying was more effective (0%, *n* = 0/39 vs. 25.6%, *n* = 23/90),^25^ while in the other study, it was less effective (25.0%, *n* = 12/48 vs. 48.2%, *n* = 13/27) compared to wiping methods^26^ (Figure 2). One study measured residual contamination as the concentration of viral RNA on surfaces shows an 88.0% reduction following wiping compared to a 15.1% reduction after disinfection by spraying (Table 5a, Table 5b, Table 5c).^25,26,27^

**TABLE 5a:** Description of type of disinfectants in included studies.

Kim et al. 2020^25^	Site A	Site B	Site C	Site D
**Setting**				
Room type	Airborne infection isolation room	Airborne infection isolation room	Isolation room without negative air pressure	Isolation room without negative air pressure
Disinfection	Before admission/after discharge	Before admission/after discharge	Before admission/after discharge	Before admission/after discharge
		Daily surface wipes containing benzalkonium chloride 0.4% and four enzymes (protease, alpha-amylase, lipase, and cellulase)		Spray disinfectant twice daily

Note: Please see the full reference list of the article Okebe J, Ewa A, Aquaisua E, et al. Disinfection methods for preventing COVID-19 infections in healthcare settings: A rapid review. J Public Health Africa. 2025;16(2), a588. https://doi.org/10.4102/jphia.v16i2.588, for more information.

**TABLE 5b:** Description of type of disinfectants in included studies.

Lesho et al. 2022^26^	Acute care hospital	Acute care hospital	Acute care hospital	Long-term care facility	Long-term care facility
**Setting**					
Room type	Mix of semi-private, shared, 48 ICU beds, 14 special care nursery bed	Mix of semi-private, shared, 48 ICU beds, 14 special care nursery bed	Mix of semi-private, shared, 48 ICU beds, 14 special care nursery bed	Shared rooms with two occupants	Shared rooms with two occupants
Disinfection	Hydrogen peroxide and peracetic acid	Hydrogen peroxide and peracetic acid	Hydrogen peroxide and peracetic acid	-	-
	Enhanced terminal cleaning involved wet mopping and surface wiping with disinfectants PLUS UV light (60 000 mJ/cm^2^)	Enhanced terminal cleaning involved wet mopping and surface wiping with disinfectants PLUS Electrostatic spray (Clorox Total 360)	Enhanced terminal cleaning involved wet mopping and surface wiping with disinfectants PLUS UV light and electrostatic spray (60 000 mJ/cm^2^ + Clorox Total 360)	Proprietary chemicals and room fogging	Proprietary chemicals and room fogging PLUS spraying

Note: Please see the full reference list of the article Okebe J, Ewa A, Aquaisua E, et al. Disinfection methods for preventing COVID-19 infections in healthcare settings: A rapid review. J Public Health Africa. 2025;16(2), a588. https://doi.org/10.4102/jphia.v16i2.588, for more information.

ICU, intensive care unit; UV, ultraviolet.

**TABLE 5c:** Description of type of disinfectants in included studies.

Lugo et al. 2021^27^	A	B	C	D
**Setting**				
Room type	Infirmary table	Keyboard in nursing area	-	Infirmary table
Disinfection	Electrolysed super-oxidation solution	Activated high-power chlorine oxidants, such as hypochlorous acid 75%, hypochlorous ion 15%, chlorine dioxide 8% and ozone 2%	UV light administered by means of equipment manufactured within the hospital itself that emits 240 nm UV rays	Wet wipes with 0.5% w/w hydrogen peroxide-based disinfectant

Note: Please see the full reference list of the article Okebe J, Ewa A, Aquaisua E, et al. Disinfection methods for preventing COVID-19 infections in healthcare settings: A rapid review. J Public Health Africa. 2025;16(2), a588. https://doi.org/10.4102/jphia.v16i2.588, for more information.

UV, ultravioloet; w/w, weight per weight.

The certainty of the evidence was rated as very low for surface decontamination (Table 6).

**TABLE 6:** Certainty of evidence assessment (grading of recommendations, assessment, development and evaluations).

Number of studies	Certainty assessment						Number of patients		Effect		Certainty	Importance
	Study design	Risk of bias	Inconsistency	Indirectness	Imprecision	Other considerations	Spraying	Wiping methods of disinfection	Relative (95% CI)	Absolute (95% CI)		
**Surface contamination (assessed with viral RNA threshold)**												
2	Observational studies	Serious^a^	Serious^b^	Not serious	Serious^b^	None	13/66 (19.7%)	35/138 (25.4%)	Not estimable	-	⨁◯◯◯ Very low	
**Concentration of viral contaminant (assessed with number of relative light units)**												
1	Observational studies	Very serious^a^	Serious^c^	Serious^c^	Serious^c^	None	†	†	†	†	⨁◯◯◯ Very low	

Note: Question: Spraying compared to wiping methods of disinfection in preventing SARS-CoV-2 infection in healthcare settings. Setting: healthcare settings providing care for COVID-19 infection.

CI, confidence interval.

† , The study reported a before and after difference in reduction in viral concentration in the spraying and wiping arm as 55 and 29 relative light units (url) respectively. This represented a 15% and 88% reduction from baseline values, respectively.

a , Potential risk of bias in outcome measurement. Insufficient detail to assess risk in domains.

b , Effect varies from lower to higher proportion of residual contamination following interventions between included studies.

c , Measured only in one study. Likely to change in different settings.

## Discussion

### Summary of main results

This review compared the evidence on the effect of disinfectant use by spraying or wiping on the reduction of the risk of SARS-CoV-2 infection in healthcare settings providing care for patients with COVID-19. Three studies met the eligibility criteria and were included for assessment. Two studies assessed the proportion of detectable viral RNA on surfaces after disinfection showed divergent results with spraying being better in one study but less effective compared to wiping, in the other. The third study that compared the concentrations of residual viral RNA showed spraying may be less effective than wiping methods. The studies could not be combined in a meta-analysis because of substantial heterogeneity. No studies report on a direct risk to human infection from contaminated surface.

Contaminated surfaces are an important source for transmitting microorganisms and IPC protocols with a focus on cleaning and disinfection of surfaces, materials and equipment play a critical role in reducing the risk of infection from such surfaces.^28^ In the wake of the COVID-19 pandemic, there was a surge in deaths and morbidity especially in healthcare settings which sparked urgent reviews to re-assess the risk of hospital-acquired infections especially from the SARS-CoV-2. In this review, eligible studies did not directly measure the risk of transmission from surfaces and materials to humans. They assessed evidence for residual contamination from both methods when applied based on standardised protocols. The authors mention adherence to cleaning protocols by healthcare staff but did not provide information on the content of these protocols or the way adherence was assessed.^26^ The studies applied a wide range of disinfectants, and the relative effect of the disinfectants would not be compared. In addition, different outcomes were used in determining residual contamination between the studies. These factors introduce substantial heterogeneity between the studies that precludes a meta-analysis for effect on transmission reduction. A systematic review of surface disinfection efficacy studies highlighted those variations in experimental conditions as an important determinant in outcomes.^36^

The effectiveness is likely to depend on additional factors such as proficiency of the personnel implementing protocols, disinfectant application mode, extent of contamination and surface type. The studies involved a combination of in-house and external cleaning staff. In practice, IPC protocols apply a combination of spraying and wiping methods and the diverse nature of the type of disinfectants and potentially cleaning protocols are likely to influence the overall effect of these methods.

It is useful to note that most of the studies did not confirm complete disinfection following the cleaning exercise. This provides evidence that current protocols may not be as efficient; however, the studies did not report any infection directly associated with residual contamination. This is a potential weakness of the studies. However, it is not clear if the residual viruses detected are viable and are a potential risk.

The included studies did not report on any actual or potential risks associated with the methods of disinfection.

### Quality of the evidence

The three included studies were substantially different in the methods applied to the interventions that preclude a direct comparison or meta-analysis. Two studies^26,27^ involved complex cleaning protocols that involved a baseline disinfection by wiping. The overall score for the risk of bias in the included studies ranged from moderate^25,26^ to serious.^27^ There were serious concerns on the lack of sufficient information to judge the potential risk of confounding from co-interventions. The certainty of the evidence was rated as very low for the effect on surface decontamination.

### Potential limitations in the review process

We have taken specific steps to limit potential bias in the review process. We applied procedures published by the Cochrane Methods Group^37^ and included the major libraries in the search strategy limited by year but not by publication status or language. The evidence reported reflects the scope of the guideline which is stated to be ‘in the context of COVID-19’ pandemic and is current till the date of the search. As evidence in this area continues to emerge, additional evidence may become available which would inform a revisit of this review’s findings. The potential risk of infection from other public areas such as schools or sports centres where contact and disinfection concerns during the COVID-19 pandemic is equally high are addressed in a separate review.

## Conclusion

Disinfection of surfaces by spraying and wiping methods in healthcare settings providing care to patients with COVID-19 may protect against SARS-CoV-2 infections indirectly by reducing residual surface contamination. This review showed variable but important reduction in residual contamination following both methods. A direct effect on SARS-CoV-2 infections could not be demonstrated because of substantial heterogeneity in the design and implementation of the included studies. Both methods are typically combined in the context of wider IPC protocols hence challenging to determine the contribution of each method.

### Implications for practice

Disinfection applied by spraying and wiping methods contribute to reducing the risk of transmission of pathogens on contaminated surfaces in regular use/contact in healthcare settings. A combination of cleaning methods and agents are likely to be beneficial in reducing the volume and proportion of infectious contaminants.

### Implications for research

There is a lot of evidence on the subject that is based on laboratory and model-based simulation studies. While these are useful in estimating the risk of surface contamination, evidence of direct risk of transmission of SARS-CoV-2 remain limited in clinical settings. The risk of transmission also depends on the cleaning practices and protocols as well as adherence by healthcare workers and patients in these settings. There remains a good case for disinfection by spraying or by wiping but the evidence on best practice needs to be carefully curated. The COVID-19 pandemic triggered a surge of research in IPC. However, the volume of potential but ineligible studies suggests important disconnections between the research and public health needs and questions that needs to be addressed.
